# The senescence difference between the central and peripheral cornea induced by sutures

**DOI:** 10.1186/s12886-023-02917-1

**Published:** 2023-04-20

**Authors:** Suxia Li, Ning Wang, Qiaoqiao Dong, Muchen Dong, Mingli Qu, Yao Wang, Weiyun Shi

**Affiliations:** 1 Shandong Eye Hospital, Shandong Eye Institute, Shandong First Medical University & Shandong Academy of Medical Sciences, Jinan, 250000 China; 2 State Key Laboratory Cultivation Base, Shandong Provincial Key Laboratory of Ophthalmology, Shandong Eye Institute, Shandong First Medical University & Shandong Academy of Medical Sciences, 5 Yan’erdao Road, Qingdao, 266071 China

**Keywords:** Senescence, Keratocyte, Fibroblast, Wound healing, Cornea

## Abstract

**Introduction:**

Cell senescence plays a regulatory role in tissue fibrosis. Corneal scarring is usually more severe in the central cornea based on clinical observation. In this study, we attempted to explore the senescence difference between the central and peripheral cornea in an in vivo mouse model with suture-induced senescence and in an in vitro model of senescence with hydrogen peroxide (H_2_O_2_)-induced rabbit corneal fibroblasts.

**Methods:**

Male Balb/c mice (6–8 weeks) received sutures in the central, superior, inferior, nasal, and temporal cornea. The sutures were removed on the 14th day. Corneal neovascularization was observed under a slit lamp microscope with a digital camera. The fibroblasts isolated from the central and peripheral rabbit cornea were induced with H_2_O_2_ to establish the senescence model in vitro. Senescence was evaluated with SA-β-gal staining and gene expression analysis of p21, p27, and p53.

**Results:**

Senescent cells accumulated in the corneal stroma from the third day to the 14th day after the operation and peaked on the 14th day. More senescent keratocytes were observed in the peripheral cornea of the mouse model. In vitro, the peripheral corneal fibroblasts were more prone to senescence due to H_2_O_2_. The polymerase chain reaction results showed that the senescence-related genes p21, p27, and p53 were highly expressed in the peripheral corneal fibroblasts compared with the central corneal fibroblasts.

**Conclusions:**

Senescent fibroblasts can limit tissue fibrosis; hence, the senescence difference between the central and peripheral cornea may contribute to the difference in scarring.

## Introduction

The cornea in the anterior part of the eye is transparent and has tissue integrity, a feature that is very important for clear vision. Keratocytes, as the main cells in the corneal stroma, usually remain stationary between the collagen layers and secrete the necessary extracellular matrix (ECM) proteins [1–3]. When eye injuries (such as trauma, infection, and inflammation) occur, corneal cells may be transformed into fibroblasts and myofibroblasts, which rapidly secrete excessive ECM protein and cause corneal scar formation during wound healing [4]. Along with keratocyte activation, new blood vessels gradually arise from the peripheral cornea to the central cornea [5]. Our previous study has reported that senescent fibroblasts can promote alkali-induced corneal neovascularization (CNV) [6]. In clinical practice, scarring is usually disk-shaped in the central cornea and more severe than that in the periphery. The recovery of corneal transparency after an injury is important for vision restoration [7].

Cellular senescence refers to the irreversible cell cycle arrest caused by cellular pressures, such as DNA damage, oncogene activation, and oxidative stress [8]. Senescent cells show a large and flattened morphology, with positive senescence-associated β-galactosidase (SA-β-gal) staining. In addition to growth arrest, senescent cells also upregulate many secreted proteins, including cytokines and growth factors, called senescence-related secretory phenotype (SASP) or senescence information secretory group (SMS) [9–11]. Aside from suppressing tumorigenesis, the senescence response participates in a variety of histopathological processes through SASP/SMS [12]. Recent reports have shown that senescent cells inhibit fibrosis of the liver, skin, and heart [12–14], indicating that cellular senescence is an effective regulator of fibrosis limitation, although this has not yet been studied in the cornea. Based on these studies, we hypothesized that the senescence of corneal fibroblasts might contribute to the difference in scarring between the central and peripheral cornea.

In this study, we examined cellular senescence in the central and peripheral cornea and found that senescent corneal fibroblasts were more frequently presented in the peripheral cornea in mice, which was in line with the results of the in vitro experiment. The senescent corneal fibroblasts obtained from the peripheral cornea exhibited a higher level of senescence-related genes p21, p27, and p53.

## Materials and methods

### Suture-induced mouse model of corneal senescence

Balb/c mice (male, 6–8 weeks; Beijing Pharmacology Institute, Beijing, China) were used for the study. All animal experiments were conducted in accordance with the Declaration on the Use of Eye and Vision Research Animals of the Vision and Ophthalmology Research Association and approved by the Ethics Committee of Shandong Institute of Ophthalmology. All animal experiments were conducted in accordance with the ARRIVE guidelines. After systemic and topical anesthesia, five interrupted stitches of the 11 − 0 polypropylene suture (Mani, Togichi, Japan) were placed in the central, superior, inferior, nasal, and temporal cornea, respectively. Ofloxacin eye ointment was immediately applied to the eye surface after the injury and once a day for one week to avoid infection. Only one eye was operated on in each mouse for all experiments. The suture was removed on the 14th day. At 12 h and 3, 5, 7, 14, and 21 days after the operation, corneal edema, neovascularization, and scarring were observed under a slit lamp microscope with a digital camera.

### Cell culture and senescence induction

New Zealand white rabbits (male, 2–4 months; Kangda, Qingdao, Shandong, China) were used for corneal fibroblast culture. The peripheral and central corneal tissues were isolated using a trephine of 8 mm and 5 mm diameter, respectively. The corneal epithelium and endothelium were digested with 2.4 U/ml dispase II (Roche, Basel, Switzerland) overnight at 4 °C. The corneal stroma was cut into pieces and incubated with 2 mg/ml collagenase I (Invitrogen, Carlsbad, CA) for 2–4 h at 37 °C. The cells were then collected and cultured in DMEM/F-12 medium supplemented with 10% FBS. A hydrogen peroxide (H_2_O_2_)-induced in vitro model of senescent corneal fibroblasts was established as previously described. Briefly, the cells were exposed three times to 200 µM H_2_O_2_ for 1 h daily. After each treatment, the cells were washed with PBS and incubated in a complete medium for 24 h.

### Senescent cell assay

The mouse eyeballs were embedded in the Tissue-Tek optimum cutting temperature compound, and 8-µm sections were created (n = 5). The cryosections were fixed with ice methanol at − 20 °C for 10 min. The rabbit keratocytes were fixed with 4% paraformaldehyde at room temperature for 10 min. Cellular senescence was identified by SA-β-gal staining (Beyotime, Haimen, China). In brief, the samples were washed with PBS, fixed at room temperature for 15 min, washed 2–3 times again before incubation in SA-β-gal staining solution (pH 6.0) overnight at 37 °C, and then observed under a Nikon microscope.

### Real-time quantitative PCR reaction

Total RNA was extracted from the H_2_O_2_-treated peripheral and central rabbit corneal fibroblasts using Nucleospin RNA kits (BD Biosciences, Palo Alto, CA). cDNA was acquired from the total RNA using the first-strand cDNA synthesis kit (TaKaRa, Dalian, China). Real-time PCR analysis was then performed with the SYBR Green PCR reagent (Invitrogen, Carlsbad, CA) in an Applied Biosystems 7500 real-time PCR system (Applied Biosystems, Foster City, CA). The cycling system was an initial denaturation cycle at 95 °C for 10 s, followed by 45 cycles at 95 °C for 15 s and 60 °C for 1 min. The results were assayed by the comparative threshold method (2^−ΔΔCt^). The nucleotide sequences of primers used in this assay are listed in Table 1. GAPDH was used as an endogenous control gene.

**Table 1:** Nucleotide sequences of primers used for RT-qPCR

Genes	Forward primer	Reverse primer	Gene accession
P21	AGCGCTGGAACTTCGACTTT	TGAGGTGCTCGGCCGCTT	XM_002714669.3
P27	CCGCCTGCAGAAACCTCTT	CCACCTCCTGCCATTCGTA	XM_017343370.1
P53	TACTCCCCCTGCCTCAACAA	GCGTCTGACAACTTCCGTCAT	NM_001082404.1
GAPDH	CGCCTGGAGAAAGCTGCTAA	CCCCAGCATCGAAGGTAGAG	NM_001082253.1

### Statistical analysis

The data in this study were representative of more than three different experiments and presented as mean ± SEM. We have analysed the images using imageJ software.The differences between the control and treated groups were compared with the Student’s t-test. P < 0.05 was considered significant.

## Results

### Corneal neovascularization after suturing

**Fig. 1:**
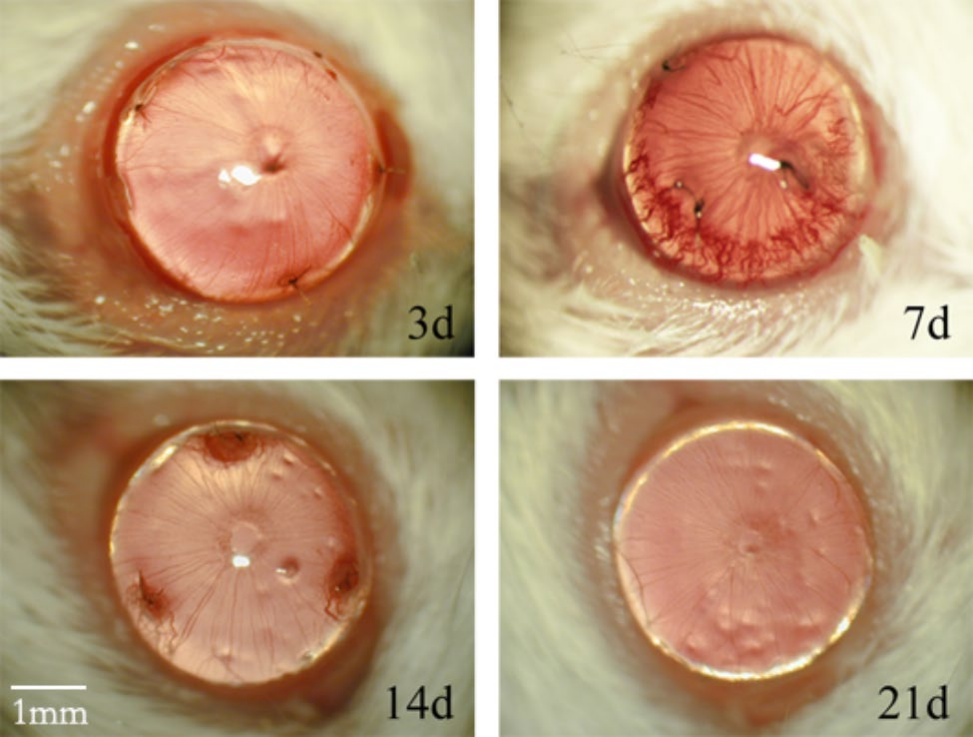
Cornea neovascularization appeared after suturing. CNV appeared on the third day, peaked on the seventh day, and subsided around the suture on the 14th day. After the sutures were removed on the 14th day, the new blood vessels degenerated rapidly and could not be observed on the 21st day (n = 3)

Corneal neovascularization was detected in the suture-induced senescence mouse model. The new vessels grew gradually from the limbal region to the central cornea after sutures were placed on the cornea. They emerged on the third day, became dense and rough on the seventh day, and faded to the surrounding sutures on the 14th day. After the sutures were removed on the 14th day, the new vessels regressed rapidly and could not be observed on the third and 21st days (Fig. 1).

### Senescent cell accumulation in the corneal suture model

**Fig. 2:**
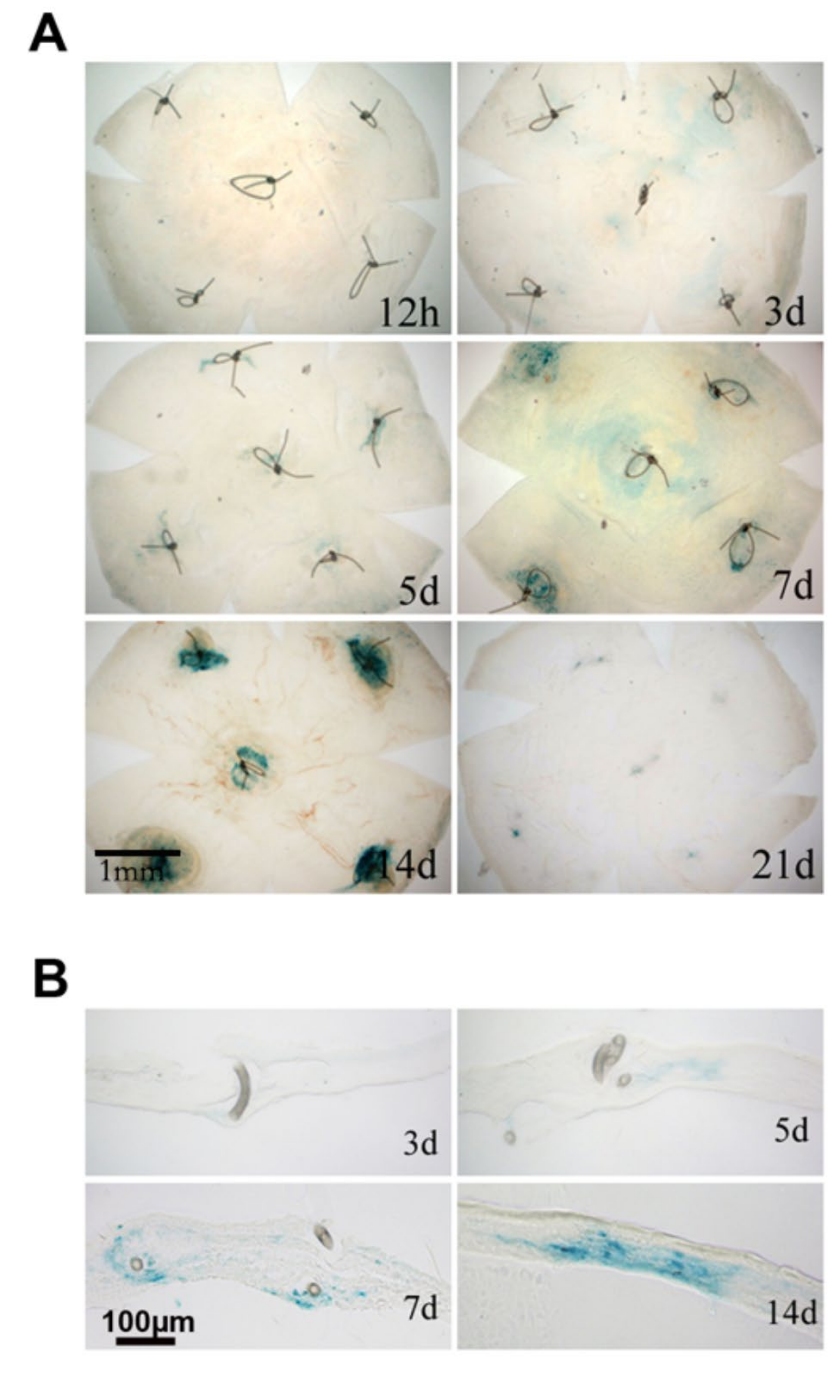
The senescent cells were stained blue by SA-β-gal in the cornea after suture induction. (A) Mild staining of SA-β-gal activity was detected in the cornea on the third day. Strong positive staining was consistently observed on the fifth and seventh days and peaked on the 14th day. The suture was removed on the 14th day, and the senescent cells completely disappeared on the 21st day (n = 3). (B) The senescent cells were mainly localized in the corneal stroma (n = 3)

In the mouse model of senescence induced by localized corneal sutures, SA-β-gal staining revealed the senescent cell distribution on the whole corneal mount. No positive SA-β-gal staining was observed in the cornea 12 h after the operation. Mild staining of SA-β-gal activity was detected in the cornea on the third day, and strong positive staining was consistently observed on the fifth and seventh days, peaking on the 14th day. After suture removal on the 14th day, the SA-β-gal staining weakened rapidly until the senescent cells disappeared completely on the 21st day (Fig. 2A). The SA-β-gal staining of the corneal sections showed that the senescent cells were mainly localized in the corneal stroma, especially around the sutures, so the possibility of corneal epithelial or endothelial cell senescence was excluded (Fig. 2B).

### Senescence difference between the central and peripheral cornea

**Fig. 3:**
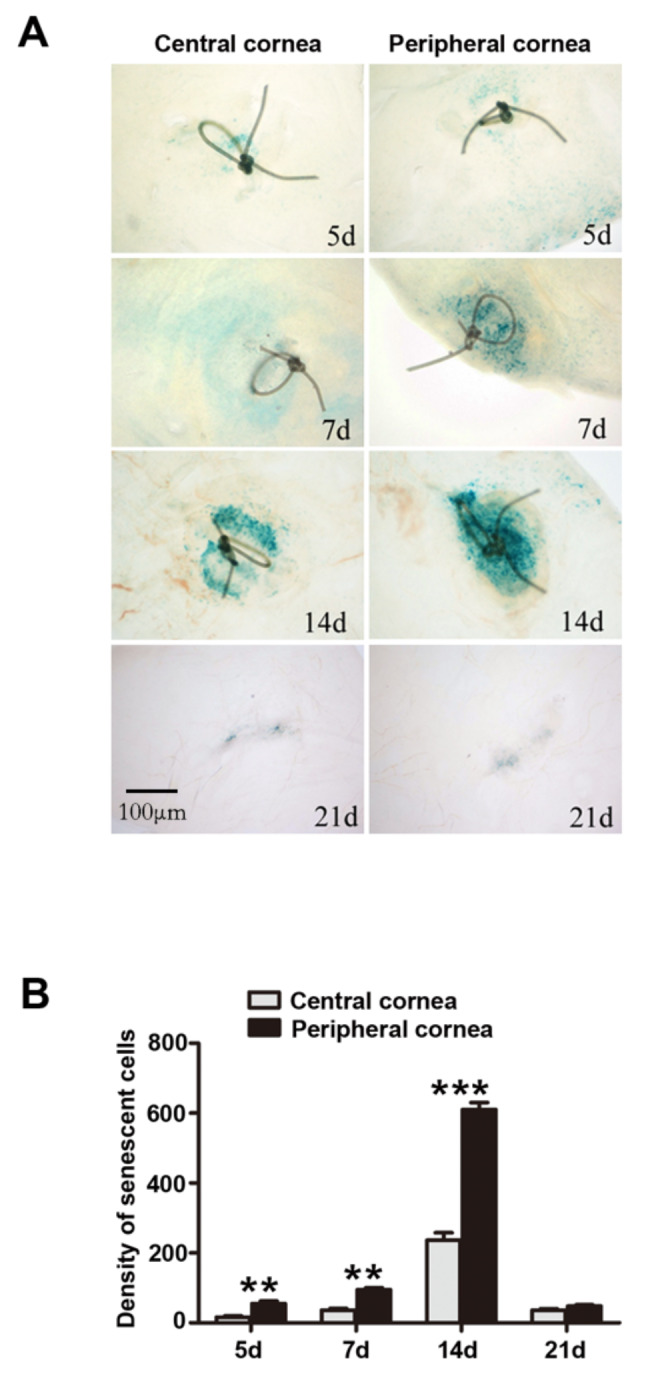
Difference in senescent cell density between the central cornea and the peripheral cornea. (A) At each time point after the suturing operation, more senescent cells were found in the peripheral cornea than in the central cornea (n = 3). (B) The density of senescent cells in the central and peripheral cornea. *P < 0.05, versus the central cornea group. Data are shown as means ± SEM (n = 3)

To compare the relative percentage of senescent cells in the central and peripheral cornea, we placed sutures at five different regions (Fig. 2A). The whole flat mount that contained tissues from both the central and peripheral suture regions was prepared for SA-β-gal staining. The staining was graded by optical density measured through the Image J software. On the fifth day after the operation, positive senescence expression was found in all five locations. The density of SA-β-gal staining positivity was higher in the peripheral area than in the central area (Fig. 3A). The findings were consistent at different time points, and the most significant difference appeared on the 14th day (Fig. 3).

### Induction of senescent corneal fibroblasts in vitro

**Fig. 4:**
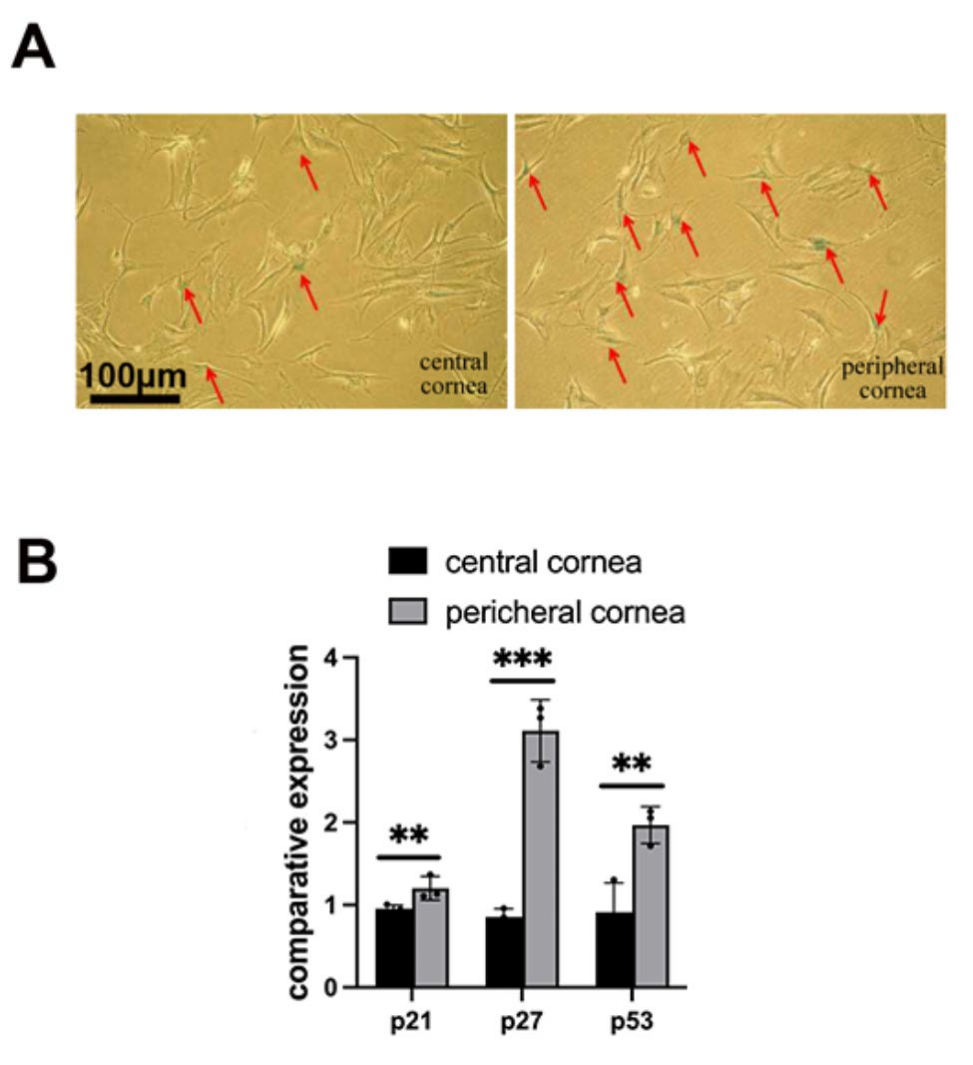
The senescent difference between peripheral and central corneal fibroblasts induced by H_2_O_2_. (A) The rabbit corneal fibroblasts from the peripheral cornea assumed more positive senescence-associated β-galactosidase staining (red arrow) (n = 3). (B) The peripheral corneal fibroblasts showed higher levels of senescence-related genes p21, p27, and p53 after induction by H_2_O_2_. *P < 0.05, versus the central cornea group. Data are shown as means ± SEM (n = 3)

To further understand the difference in the senescence ability between the central and peripheral corneal fibroblasts, rabbit corneal fibroblasts were obtained and induced to senescence in vitro with H_2_O_2_. SA-β-gal staining was used to identify cell senescence. The senescent fibroblasts were found to be enlarged and flattened with accumulated SA-β-gal staining. Similar to the in vivo experimental results of the mouse corneal model, under the same experimental conditions, the density of the senescent cells in the peripheral cornea was much higher than that in the central cornea (Fig. 4A). Real-time RT-PCR results showed that the levels of senescence-related genes p21, p27, and p53 in the peripheral corneal fibroblasts were higher than those in the central fibroblasts, which also proved that peripheral corneal fibroblasts were more prone to senescent (Fig. 4B).

## Discussion

In the present study, we observed that in the mouse model of cellular senescence induced by corneal sutures, the senescent corneal fibroblasts were more frequently found in the peripheral cornea than in the central cornea. In vitro, the rabbit corneal fibroblasts from the peripheral cornea were more likely to have senescence-associated β-galactosidase staining, and the senescence-related gene level was higher in the peripheral corneal fibroblasts. Since senescent fibroblasts can act as important regulators of tissue fibrogenesis [13], we believe the senescence difference was one of the reasons for corneal scar formation after trauma, infection, or inflammation.

Eye trauma, such as sutures on the cornea, may cause serious damage during wound healing, such as corneal matrix turbidity and new blood vessels [15–17]. Our previous studies have described that senescent cells from activated corneal fibroblasts can promote alkali-induced CNV [6]. In this study, senescent fibroblasts were also found to participate in CNV, although they appeared later than the development of CNV.

During wound healing, quiescent keratocytes were activated by inflammation-induced transforming growth factor β and transformed into fibroblasts and myofibroblasts, which rapidly synthesized and secreted redundant ECM proteins and repaired the wound [18]. After the completion of wound repair, myofibroblasts become senescent or transform to scar keratocytes [19]. Recent studies have shown that one of the roles of senescent fibroblasts in tissue repair is to limit fibrosis, which is usually observed in chronic wounds. The mark of cellular senescence is cell cycle arrest, and the enforced cell cycle arrest of senescent fibroblasts in vivo slows the fibrogenic response to damage by limiting the expansion of the cell type responsible for producing the fibrotic scar [14]. A number of MMPs, including MMP2, MMP3, and MMP9, are part of the SASP, which can degrade excessive collagen and maintain tissue homeostasis during wound healing [20]. The stable cell cycle arrest of aging fibroblasts and SASP may also be a mechanism for corneal scarring.

The central cornea of keratoconus is thinner than the peripheral cornea, and the central cornea is more prone to scar formation, underscoring a difference between the central and peripheral corneal cells [21]. Our results showed that the levels of senescence-related genes p21, p27, and p53 were higher in the peripheral cells. The expression of p21 and p27 is strictly controlled by the tumor suppressor protein p53, which plays a role in cell apoptosis, senescence, genomic stability, and inhibition of angiogenesis [22]. P21 maintains the cell cycle at the G1/S stage and arrests growth. The higher expression of these genes may partially contribute to peripheral keratocyte senescence.

## Conclusion

Our study indicates that the senescence difference between the central and peripheral cornea may contribute to the disparity in scarring, as senescent fibroblasts can limit tissue fibrosis.

## Data Availability

All data generated or analyzed during this study are included in this article and its supplementary material files. Further inquiries can be directed to the corresponding author.
